# All solid state rechargeable aluminum–air battery with deep eutectic solvent based electrolyte and suppression of byproducts formation

**DOI:** 10.1039/c9ra04567h

**Published:** 2019-07-17

**Authors:** Ryohei Mori

**Affiliations:** Green Science Alliance Co., Ltd. 2-22-11 Obana Kawanishi City Hyogo Prefecture 666-0015 Japan; Fuji Pigment Co. Ltd 2-23-2 Obana Kawanishi City Hyogo Prefecture 666-0015 Japan moriryohei@fuji-pigment.co.jp +81-72-759-9008 +81-72-759-8501

## Abstract

In order to create a rechargeable aluminum (Al)–air battery, an aluminum–air battery with a deep eutectic solvent-based solid electrolyte was prepared. The prepared battery demonstrated a capacity smaller than the theoretical value although we observed stable electrochemical reactions. When TiN was used as an air cathode material, byproducts of the aluminum–air battery such as Al(OH)_3_ and Al_2_O_3_ were not detected on either the Al anode nor the air cathode. Even though we did not detect byproducts, we observed NaCl and NH_4_Cl phases on the air cathode, and they did not hinder the electrochemical reaction.

## Abstract 2

In order to create a rechargeable aluminum (Al)–air battery, an aluminum–air battery with a deep eutectic solvent-based solid electrolyte was prepared.

## Graphical abstract

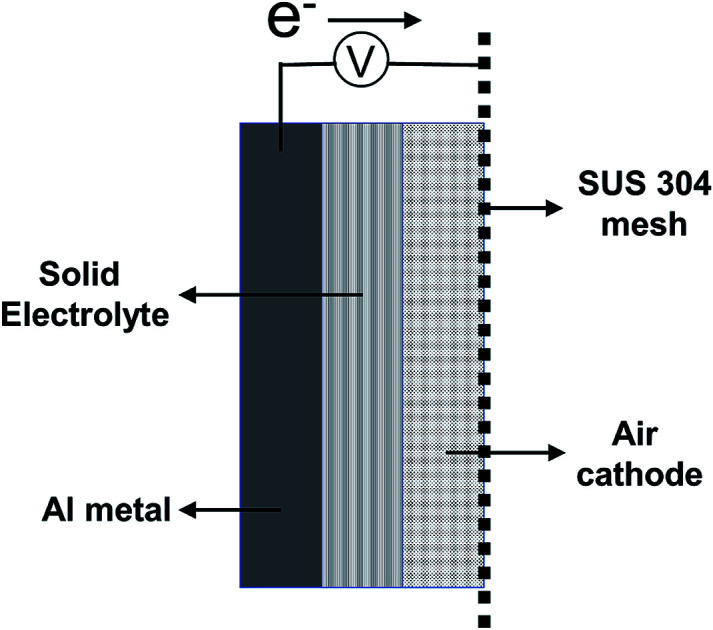

## Introduction

Advanced materials that permit the efficient harvesting, storage, and utilization of renewable energy are at the heart of ongoing research in the energy field.^1–4^ To date, Li ion batteries are the most successful energy-storage solution; they have been widely used in both portable electronics and electric vehicles (EVs) since the first report was made on them in 1991. Unfortunately, their limitations of high cost, insufficient energy density, and unsatisfactory safety have prevented their large-scale applications in the automobile industry, especially for extended-range EVs.^5,6^ In this regard, many post-lithium-ion technologies, including Li–S,^7^ Na ion,^8^ all-solid-state Li ion batteries,^9^ and metal–air batteries, have been proposed and actively studied.^10,11^ Recently, metal–air batteries have become a promising power source because of their high theoretical energy density and their use of atmospheric oxygen as fuel.^12–15^ Among various metal–air batteries, alkaline metal–air batteries, especially lithium–oxygen, have been intensively investigated because of their high specific energy density of up to 5200 W h kg^−1^. However, the rechargeability, safety, and cost of these batteries make them difficult to commercialize. In addition, lithium is very sensitive to ambient conditions, such as humidity and oxygen, and it is a scarce natural resource in some regions.^16,17^

Meanwhile, aluminum (Al) is inexpensive, safe, and is the third most abundant element in the Earth's crust. An aluminum-based redox couple, which involves a three-electron transfer that occurs during the electrochemical charge/discharge reactions, provides a storage capacity that rivals that of the single-electron lithium-ion battery. Its relatively low atomic weight of 26.98 and trivalent state confer it a gram-equivalent weight of 8.99 and an electrochemical equivalence of 2.98 A h g^−1^, as compared with 3.86 A h g^−1^ for lithium. Because of its lower reactivity, easier handling, and greater safety, such an Al-based battery may offer significant cost savings and safety improvements over Li ion batteries. Consequently, the use of Al as anode in metal–air batteries has long attracted attention because of its high theoretical ampere hour capacity and overall specific energy. In addition, Al is the most recycled metal on the planet and is economically cheap compared with lithium, zinc, and magnesium. A major barrier preventing the commercialization of Al-based batteries is the high rate of aluminum self-corrosion in alkaline solutions under both open-circuit and discharge conditions. Furthermore, byproducts such as Al_2_O_3_ and Al(OH)_3_ accumulate at both the anode and cathode, which also suppress electrochemical reactions.^18–22^ A number of studies have been reported regarding aluminum–air batteries using aqueous, organic solvents, and ionic-liquid-based electrolytes, and some of these have been shown to exhibit rechargeable properties. In particular, when an ionic liquid-based electrolyte was used, the aluminum–air battery began to demonstrate rechargeable battery behavior.^23–29^ An ionic analog electrolyte based on AlCl_3_ and urea has been developed as a deep-eutectic-based electrolyte, and it has been found to exhibit rechargeable behavior and a cost advantage over ionic liquid-based electrolytes.^30,31^ However, when one considers creating an aluminum–air battery for practical use, a solid-state battery is desirable because of its toughness and ease of manufacturing, which may also result in a low-cost battery. Furthermore, ionic-liquid-based electrolytes that are used should be non-volatile although we have observed that ionic-liquid-based electrolytes evaporate completely after a few months even under an ambient atmosphere at room temperature.

In this study, we showed that a solid-state, rechargeable aluminum–air battery with stable electrochemical reactions could be achieved by mixing AlCl_3_, urea, carboxymethyl cellulose (CMC), and glycerin for use as an electrolyte.

## Experimental

**Fig. 1:**
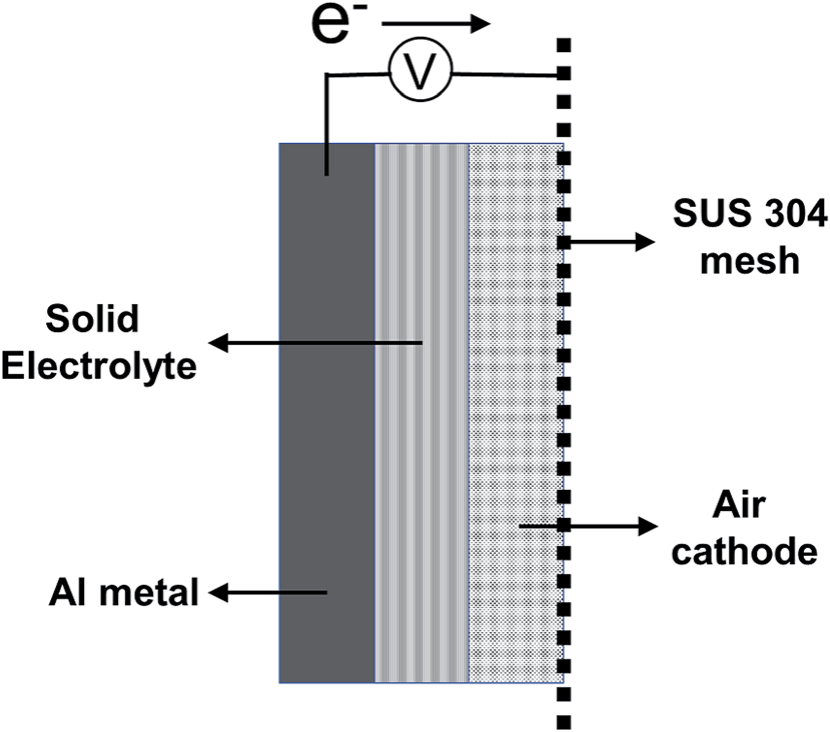
Schematic figure of Al–air battery prepared in this study.

An aluminum (Al) board (A1050, 99.5% purity) was used as an anode. To prepare the air cathode, titanium nitride (TiN) and polyvinylidene difluoride (PVDF) at 1 : 0.3 molar ratio were mixed, compressed with a pelletizer at 30 MPa, and used as a pellet-shaped air cathode. SUS 304 mesh was used as the electrical-current collector. AlCl_3_, urea, CMC, and glycerin at 3 : 2 : 1 : 1 molar ratio were mixed, and commercial gauze was soaked in this mixture for use as a solid electrolyte. For the preparation of the solid-state aluminum–air battery, the aluminum (Al) board, solid electrolyte, air cathode, and SUS 304 mesh were assembled in that order and clamped using a plastic clip. A schematic figure of the aluminum–air battery prepared in this study is shown in Fig. 1. All of the aforementioned chemicals were purchased from Sigma Aldrich Corporation (Saint Louis, USA). The electrochemical performance of the battery was evaluated by using a galvanostat (SP-150; BioLogic, France). The measured area of the prepared aluminum–air battery was 1 cm^2^ for both the anode and air cathode. Cyclic voltammetry was carried out at a scan rate of 10 mV s^−1^. All electrochemical measurements were made under ambient atmospheric conditions at 25 °C and 40% humidity. Crystalline phases of the anode and air cathode were studied by X-ray diffraction (XRD) on a RAD-RU diffractometer (Rigaku Corp., Tokyo, Japan) using Cu Kα radiation at 40 kV and 200 mA. X-ray photoelectron spectroscopy (XPS) measurements were carried out on a PHI5000 Versa Probe II spectrometer (Ulvac-Phi Inc. MN, USA). The morphology and energy-dispersive X-ray spectroscopy (EDS) analysis of the air cathode was performed using a field emission scanning electron microscope (JSM-7610F, JEOL Ltd., Japan) with an acceleration voltage of 15 kV.

## Results and discussion

**Fig. 2:**
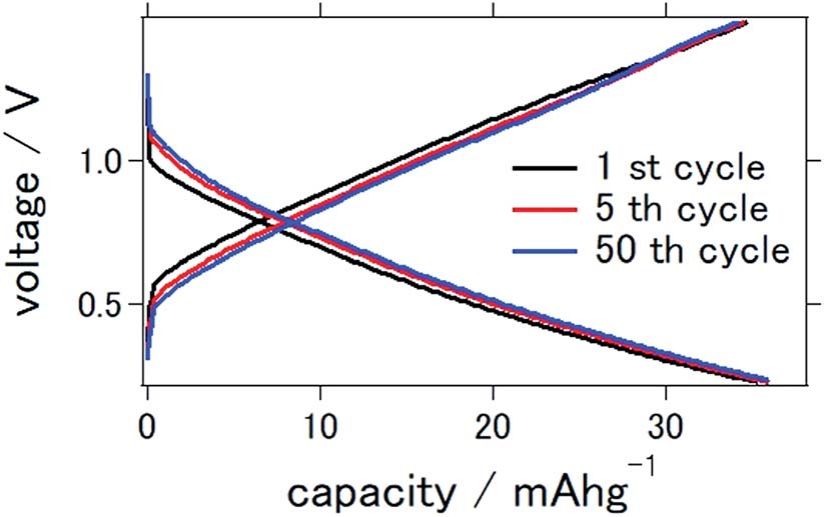
Charge–discharge curves of the all solid state Al–air battery with TiN air cathode prepared in this study.

Fig. 2 presents the charge–discharge curve of the prepared solid-state aluminum–air battery at an applied current of 0.1 mA cm^−2^, for which the cutoff voltage was 0.2–1.5 V. Although the capacity was as small as 35.8 mA h g^−1^ at the first cycle, it remained at 35.0 mA h g^−1^ even after 50 charge–discharge cycles, indicating that 97.8% of the initial capacity was maintained. It is clear that although the capacity was smaller than the theoretical value, it was stable even after a large number of charge–discharge electrochemical reactions.

**Fig. 3:**
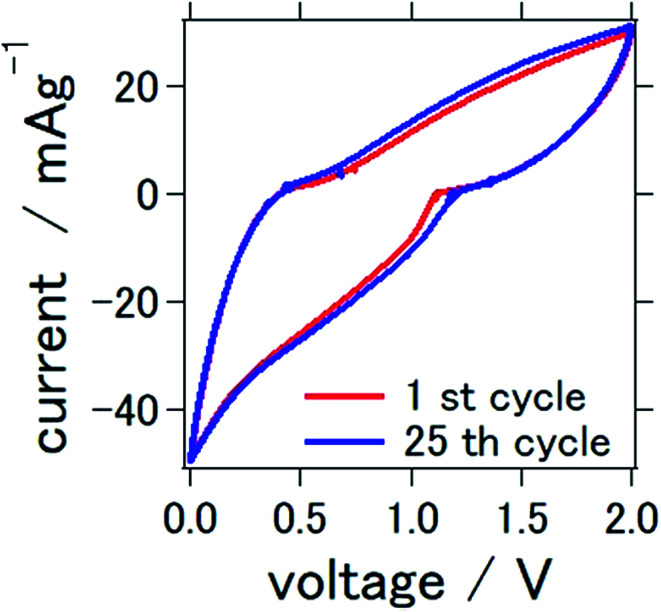
Cyclic voltammetry of the all solid state Al–air battery with TiN air cathode prepared in this study.

The cyclic voltammograms of the prepared solid-state aluminum–air battery at the first and 25th cycles are presented in Fig. 3. The cycle was measured between 0 and 2.0 V, which were used to characterize the redox reactions. Conspicuous anodic or cathodic peaks were not observed, although a stable cyclic voltammetry confirming the stability of TiN as catalytic air cathode materials was observed over repeated cycles. These results and the profile of the cyclic voltammogram were similar to those found in our previous study, in which TiN was also used as an air cathode material for an aluminum–air battery. In our previous study, we used a mixture of 1-butyl-3-methylimidazolium chloride and AlCl_3_ as an electrolyte. It is interesting to note that ionic-liquid-based electrolytes and deep-eutectic solvent-based electrolytes demonstrated similar cyclic voltammetry profiles, which suggest a resemblance of the electrochemical reactions of these two types of electrolytes.

Chloroaluminate melts are well known for their high affinity to aluminum electrodeposition and have been considered as possible electrolytes for the development of secondary Al ion batteries.^32–35^ This type of electrolyte can be basic, neutral, or acidic depending on the molar ratio of AlCl_3_ that is used. In basic melts, both AlCl_4_^−^ and Cl^−^ species coexist, whereas in neutral melts, the only anionic species is AlCl_4_^−^. In acidic melts, the predominant species is Al_2_Cl_7_^−^, and the Al deposition/stripping process is as follows:4Al_2_Cl_7_^−^ + 3e^−^ ↔ Al + 7AlCl_4_^−^

In this case, this electrochemical reaction is reversible and is said to be utilizable for a rechargeable aluminum–air battery^36^

The mechanism of the ORR/OER reaction in ionic liquid-based electrolytes remains complex, however, and it has not yet been completely elucidated. Recently, some urea- and acetamide-based deep-eutectic solvents developed by Abood *et al.* have been shown to present appropriate reversible activity for Al deposition/stripping.^37^ Here, the amide group reacts with AlCl_3_ and creates a positively charged complex and negatively charged tetrachloroaluminate anion:2AlCl_3_ + *n*Amide ↔ [AlCl_2_·*n*Amide]^+^ + AlCl_4_^−^

Even with the above descriptions, we are not sure at this stage what is the exact ion species or charge carrier for our aluminum–air battery system, although we can assume that it is AlCl_4_^−^ or Al_2_Cl_7_^−^. Katayama *et al.* stated that in the case of the aluminum–air battery with an electrolyte composed of a mixture of AlCl_3_, 1-butyl-3-methylimidazolium chloride, and bis(trifluoromethylsulfonyl)amide, the charge carrier should be either AlCl_4_^−^ or Al_2_Cl_7_^−^. They claim that when the concentration of Al_2_Cl_7_^−^ decreases, the concentration of AlCl_4_^−^ increases; this was confirmed by Raman spectroscopy.^38^ Agiorgousis *et al.* excluded the possibility of the Al^3+^ cation as the charge carrier and instead presented the AlCl_4_^−^ anion as the possible charge carrier.^39^ Furthermore, Angell *et al.* claimed that both Al_2_Cl_7_^−^ and AlCl_4_^−^ exist in the AlCl_3_^−^ urea ionic liquid analog electrolyte.^30^ Since the electrolyte contains other additives in our aluminum–air battery system such that the electrochemical mechanism may be even more complicated than the aforementioned systems, it should be further investigated in future studies. The capacity of our rechargeable aluminum–air battery is lower than the theoretical value proposed for an aluminum–air battery. This may be mainly due to insufficient contact between the electrode and solid electrolyte, as well as our non-ideal battery preparation technique.

Additionally, we could not exclude the possibility that the small observed capacity is due to the fact that our current battery system is not wholly an aluminum–air battery, but also partly an aluminum ion battery. We infer that the pellet-shaped air cathode that was prepared by compressing powder with quite strong force is very much tightly pressed. So that the ambient air might not penetrate into entire air cathode sufficiently in order to react with the charge carrier that is provided by the electrolyte. Measuring the electrochemical properties in a N_2_ atmosphere could help understand this mechanism. By the way, it should be noted here that besides the use of AlCl_3_ and urea as an electrolyte, CMC was added as a viscosity thickener to prepare a solidified electrolyte. In addition, the authors and Chen *et al.* have reported that deep eutectic solvents are volatile.^40^ Therefore, glycerin was added as a humectant for the solid-state electrolyte in our battery system. The AlCl_3_/urea/CMC/glycerin molar ratio was 3 : 2 : 1 : 1, since this components ratio demonstrated suitable viscosity for a solid electrolyte. However, more appropriate component ratios may be elucidated in further studies to enhance battery performance. It should be mentioned here that the deep-eutectic solvent-based electrolyte was quite stable because its electrochemical properties did not deteriorate even after 1 month following battery preparation (data not shown).

**Fig. 4:**
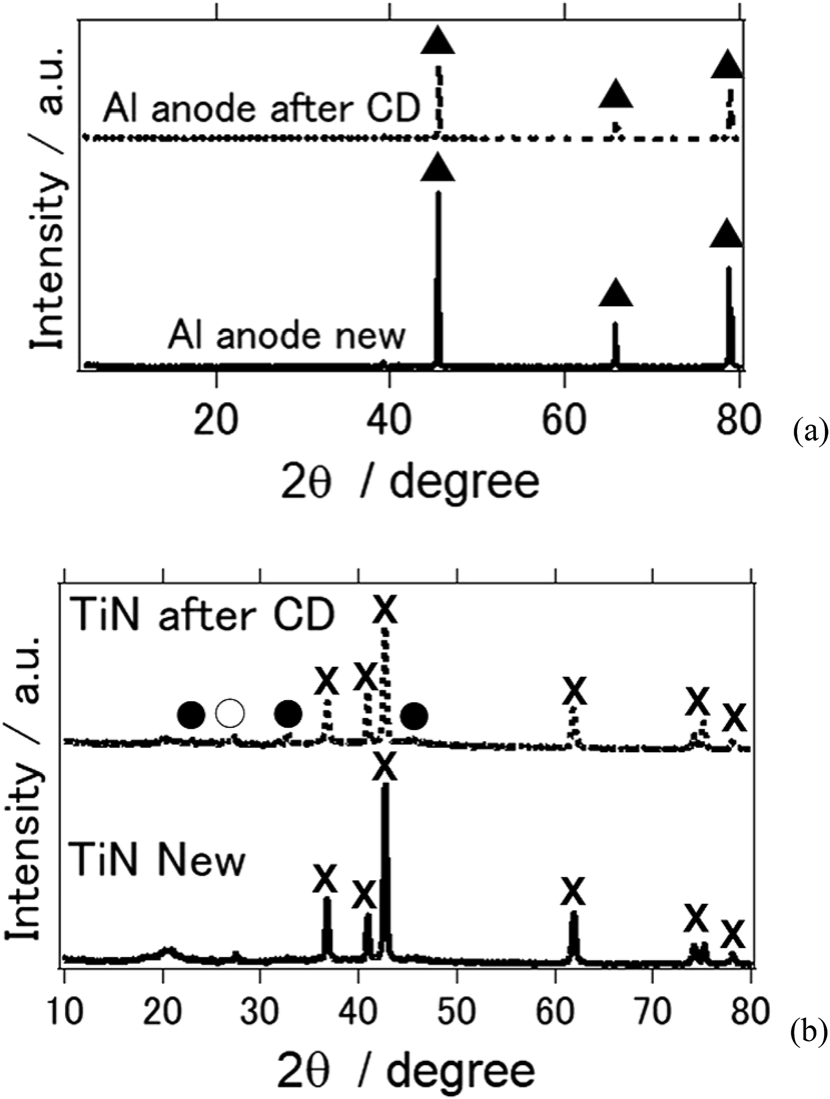
X-ray diffraction patterns of the all solid state Al–air battery (a) Al anode (b) TiN air cathode before and after the electrochemical reaction. (▲: Al metal, ×: TiN, ●: NH_4_Cl, ○: NaCl).

Fig. 4 shows the X-ray diffraction patterns of the Al anode and air cathode before and after the electrochemical reaction. CD denotes the charge–discharge electrochemical reaction. Fig. 4(a) shows the XRD of the Al anode before and after the electrochemical reaction. As can be observed, it is clear that the byproducts Al(OH)_3_ and Al_2_O_3_ were not observed. This phenomenon is similar to what was shown in previous studies in which Al deposition was possible at room temperature when an ionic-liquid-based electrolyte was used instead of an aqueous electrolyte.^26–28^ In addition, when TiN was used as an air cathode, the byproducts of the aluminum–air battery, such as Al(OH)_3_ and Al_2_O_3_, were not observed (Fig. 4(b)). This agrees with our previous study in which TiN was applied as an air cathode material and butyl methyl imidazolium chloride was used as an ionic-liquid electrolyte.^27^ It is interesting to note that when a deep eutectic solvent was used as an electrolyte, similar results were also obtained. As far as we know, this study confirms for the first time that byproducts are not observed on either the Al anode nor the air cathode when a deep-eutectic solvent-based electrolyte is used in place of an ionic-liquid-based electrolyte. It should be noted that we observed NaCl and NH_4_Cl phases, which are suggested to have formed as a result of the electrochemical reaction in our aluminum–air battery system.

**Fig. 5:**
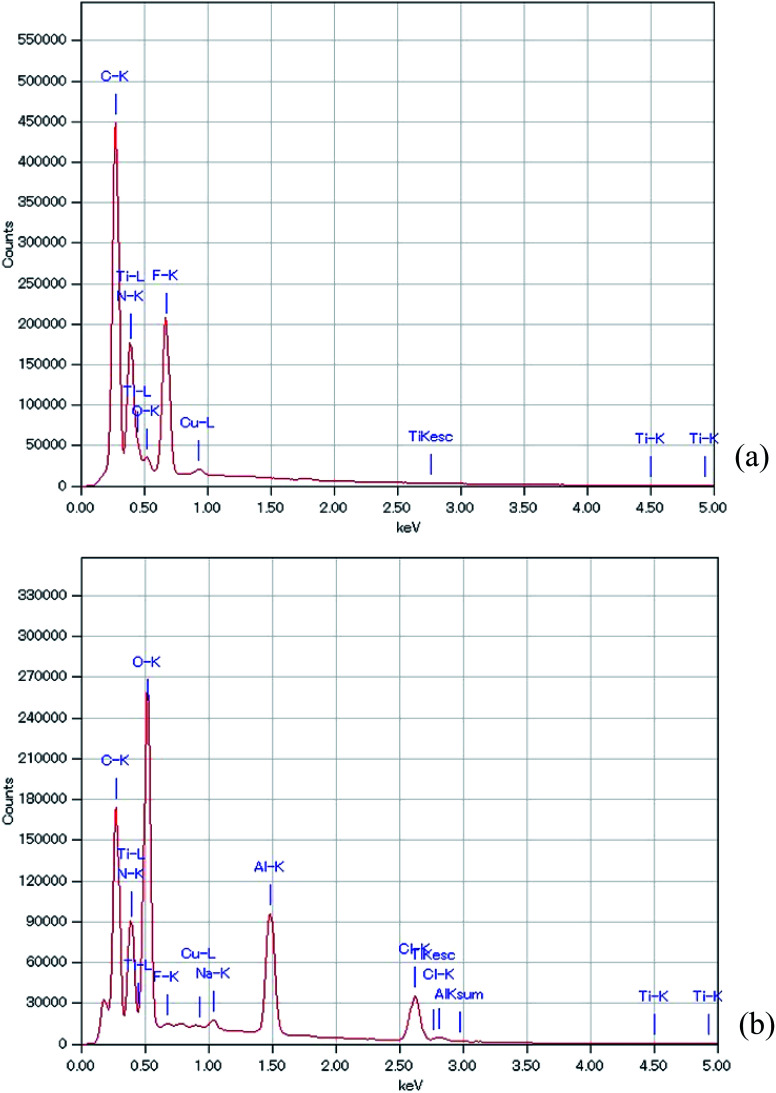
EDS of the all solid state Al–air battery TiN air cathode prepared in this study. (a) Intact TiN (b) TiN after the electrochemical reaction.

Fig. 5 shows the EDS analysis of the air cathode surface before and after the electrochemical reaction. Table 1 summarizes the data of the EDS analysis at two points to ensure the accuracy of this measurement. It should be noted here that the obtained EDS results were similar between the two points on the air cathode surface, which confirms the accuracy of our measurement. C, N, F, and Ti were the main components detected at the intact TiN surface. C, N, and F can be considered to originate from TiN itself and the binder, which is composed of PVDF, while Ti comes from TiN. After the electrochemical reaction, Ti could not be detected and the quantity of F decreased. It is inferred that some sediment covers the surface of the TiN. Instead Al, Cl, and a trace amount of Na were observed. Na and Cl could have resulted from NaCl and NH_4_Cl, as we have seen in the above XRD experiments. Furthermore, it is deduced that even though Al(OH)_3_ could not be detected by XRD, a trace amount of Al that originates from the byproducts of the electrochemical reaction was detected *via* EDS analysis. This coincides with our previous study in which we also detected trace amounts of Al in our XPS measurement.^27^

**Table 1:** Summarized EDS data of the all solid state Al–air battery TiN air cathode at 2 points, prepared in this study. (a) Intact TiN (b) TiN after the electrochemical reaction

Sample	TiN new	TiN new	TiN after CD	TiN after CD
Spot	Spot-1	Spot-2	Spot-1	Spot-2
C	27.98	28.75	14.68	23.56
N	23.35	22.85	20.63	25.19
O	2.89	2.87	25.43	30.73
F	19.86	20.06	0.52	0.14
Na	ND	ND	0.79	0.56
Al	ND	ND	12.25	8.25
Cl	ND	ND	25.46	11.52
Ti	25.7	25.24	ND	ND

**Fig. 6:**
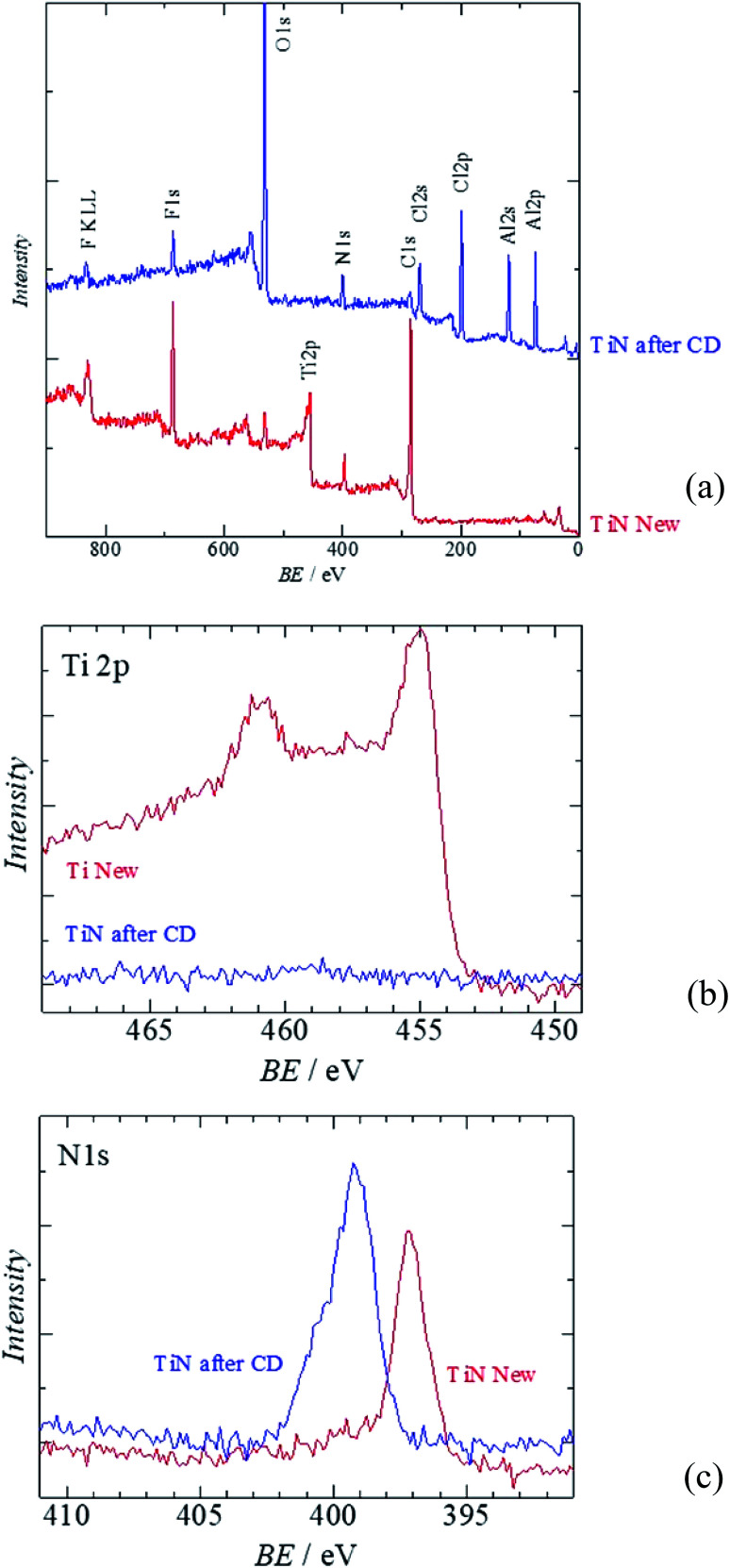
XPS of the all solid state Al–air battery TiN air cathode prepared in this study. (a) Wide scan XPS (b) Al 2p (c) Cl 2p.

Fig. 6 shows the XPS analysis of the surface of the TiN air cathode before and after the electrochemical reaction. Note that CD indicates the charge–discharge electrochemical reaction as in the XRD section. Fig. 6(a) is the wide-range spectra of TiN before and after the electrochemical reaction. Fig. 6(b) and (c) are the XPS results for Ti 2p and N 1s, respectively. Obvious differences are noted in the disappearance of the Ti 2p peak and the emergence of Al 2p and Cl 2p peaks. As we observed with the EDS analysis, the Ti atomic orbital could not be seen in the XPS measurement either. This result also shows that the surface of the TiN air cathode is covered with other sediments such NaCl and NH_4_Cl after the electrochemical reaction; this is shown by the XRD analysis. In addition, Al 2p seems to have originated from Al-collated compounds such as Al(OH)_3_ or AlCl_3_, even though these were not clearly observed from the XRD analysis. Cl 2p might have resulted from NaCl, NH_4_Cl, or AlCl_3_. This phenomenon also agrees with the EDS analysis discussed above. Furthermore, as indicated by the changes in the N 1s peaks, nitrogen exists as TiN before the electrochemical reaction. After the electrochemical reaction, the N 1s atom orbital exists as an organic compound. One of the suggested organic compounds in which this may exist is NH_4_Cl, as it was observed *via* XRD; however, it should be noted that this is only a speculation.

Both metal nitride and carbide have been reported to be excellent catalysts for the oxygen reduction and evolution reactions. It is thought that at the TiN surface, peroxide formation involves the production of an intermediate superoxide ion (O_2_˙^−^), which is followed by protonation and electron transfer to produce H_2_O_2_. However, it has also been shown that TiN may catalyze either two or four electrons for the reduction of oxygen, which is beneficial for catalytic use in metal–air batteries.^41^

2e^−^ pathwayO_2_ + 2H^+^ + 2e^−^ → 2H_2_O_2_H_2_O_2_ + 2H^+^ + 2e^−^ → 2H_2_O

Direct 4e^−^ pathwayO_2_ + 4H^+^ + 4e^−^ → 2H_2_O

It should be noted that a similar phenomenon has been observed in the case of lithium–air batteries. It has been reported that a TiC-based air cathode may be used in a lithium–air battery and that it reduces side reactions that result in electrolyte and electrode decomposition when compared with a carbon-based air cathode. In fact, they exhibited better durable battery performance with respect to the decomposition of Li_2_O_2_. They suggest that this stability may originate from the presence of TiO_2_ on the surface of TiC.^42^ We have also confirmed in our previous study that TiC and TiN did work as air cathodes for aluminum–air batteries and suppress the formation of byproducts. Thus, it could be proposed that similar byproduct formation suppression mechanisms may also occur in this deep-eutectic-based electrolyte system. It was found that some type of aluminum chemical compound was formed in the present aluminum–air battery besides byproducts such as Al(OH)_3_ or Al_2_O_3_; this compound resulted from the decomposition of the electrolyte. We believe that carbon existed as a carboxy group and not as a carbonate group, thereby suppressing the formation of Al(OH)_3_ and Al_2_O_3_.^27^ However, a detailed mechanism study of the TiC air cathode in our aluminum–air battery remains to be further investigated.

**Fig. 7:**
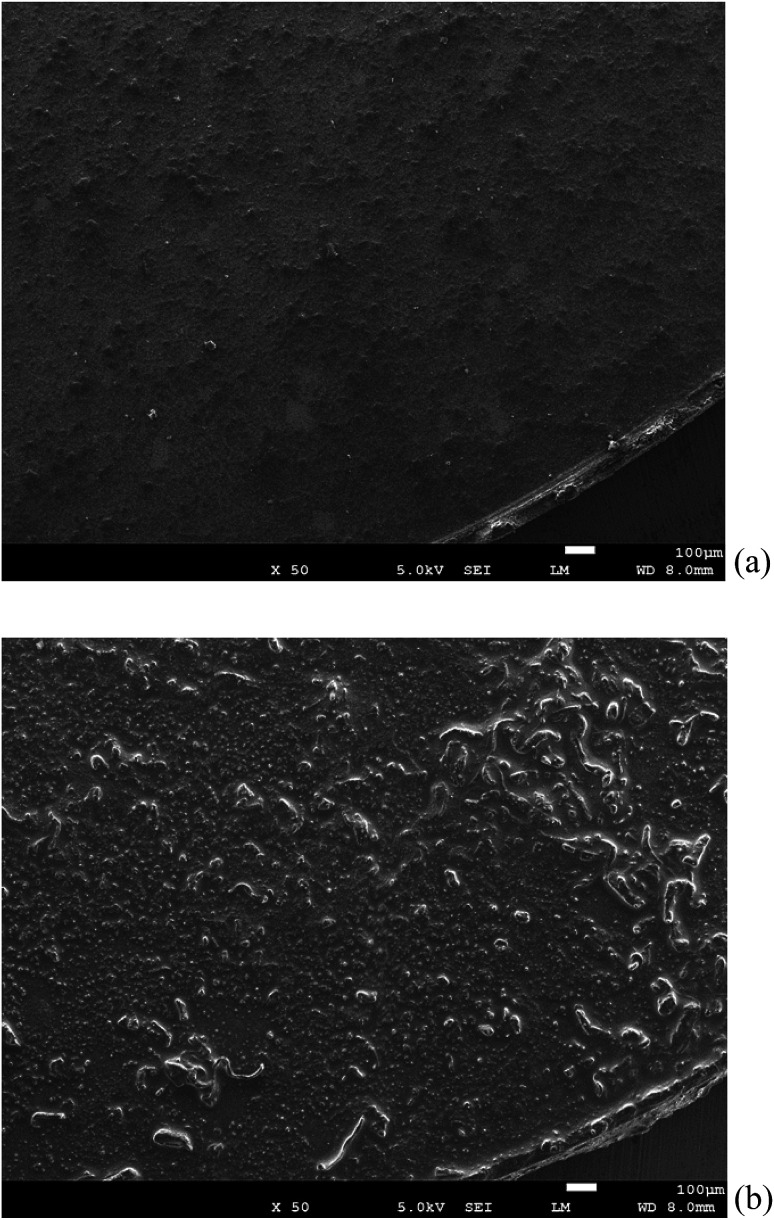
SEM observation of the all solid state Al–air battery TiN air cathode prepared in this study. (a) Intact TiN (b) TiN after the electrochemical reaction.

Fig. 7 shows the morphological observation of the surface of the TiN air cathode before and after electrochemical reaction. Fig. 7(b) presents the SEM observation of the TiN air cathode surface after the charge–discharge electrochemical reaction. As compared with Fig. 7(a), which shows the intact TiN air cathode surface, the surface is more rigid and some aggregate forms that are inferred to accumulate during the charge–discharge electrochemical reaction are visible. These aggregations and sedimentation compounds may be enriched on the surface, since we could not detect any atomic orbital of the Ti atom by EDS or XPS. We intentionally eliminated at least 5 nm of the surface layer of the TiN air cathode using an Ar^+^ ion beam before XPS measurement in order to avoid contamination effects on the measurement. This indicates that the sediments are at least thicker than 5 nm on the surface of the cathode. It is interesting to note that our aluminum–air battery is stable even after the TiN air cathode has been covered by these layers of sedimentation. It is likely that once the electrode is covered with this kind of stable layer, the aluminum–air battery becomes more stable similar to when the Li ion battery electrode is covered with the solid electrolyte interphase (SEI). This SEI is mainly composed of Li and organic chemical compounds and results from the decomposition of the electrolyte during the electrochemical reaction.^43,44^ One could suggest that this layer on the TiN air cathode could be NaCl and NH_4_Cl from our experimental results described above. From our previous study, when we applied Al_2_(WO_3_)_4_ as an internal layer between the anode and air cathode, we found that Na_2_(WO_4_)(H_2_O)_2_ formed on the air cathode after the electrochemical reaction when an aqueous NaOH electrolyte was used.^17^ This indicates that Al was replaced with Na to form Na_2_(WO_4_)(H_2_O)_2_. However, the crystalline phase would not change just by adsorbing metallic ions onto the surface of the cathode from the electrolyte. We suggest this phenomenon to be similar to the electrochemical doping procedure that Adachi *et al.* discovered previously for inorganic oxide materials.^45^ We also observed a similar phenomenon in our previous study that in addition to having Al_2_O_3_ as a byproduct of the aluminum–air battery, Na_2_Al_22_O_34_·2H_2_O was observed when aqueous NaOH was used as an electrolyte. On top of that, K_2_Al_22_O_34_ was also detected when aqueous KOH was used as an electrolyte. As Na- and K-containing complex phases appeared, a Na- and K-containing ion had been participating in the electrochemical reaction in our aluminum–air battery system.^46^ Likewise, NaCl and NH_4_Cl formed through some unknown mechanism that is unknown at this stage by replacing Al with Na. It could also be deducted that since CMC was generally partly modified with sodium, sodium was replaced with AlCl_3_ to form NaCl.

Either way, a detailed investigation must be carried out in order to create a stable, rechargeable aluminum–air battery with improved performance.

## Conclusion

A solid-state rechargeable aluminum–air battery with a solid electrolyte composed of AlCl_3_, urea, CMC, and glycerin was fabricated. The battery exhibited stable electrochemical reactions as confirmed by the charge–discharge curves and cyclic voltammogram. When TiN was used as an air cathode material, the typical byproducts of an aluminum–air battery such as Al(OH)_3_ and Al_2_O_3_ were not observed even when a deep-eutectic solvent-based electrolyte was used as an electrolyte. As far as we know, this is the first time that byproducts have not been observed on either the Al anode or the air cathode when a deep-eutectic solvent-based electrolyte was used. EDS, XPS, and SEM analysis revealed that the TiN air cathode surface was covered with a layer that did not hinder the electrochemical reaction.

## Conflicts of interest

There are no conflicts to declare.

## Supplementary Material
